# Main Olfactory and Vomeronasal Epithelium Are Differently Affected in Niemann-Pick Disease Type C1

**DOI:** 10.3390/ijms19113563

**Published:** 2018-11-12

**Authors:** Martin Witt, René Thiemer, Anja Meyer, Oliver Schmitt, Andreas Wree

**Affiliations:** Department of Anatomy, University of Rostock, 18057 Rostock, Germany; rene-thiemer@gmx.de (R.T.); anja.meyer2@uni-rostock.de (A.M.); oliver.schmitt@med.uni-rostock.de (O.S.); andreas.wree@med.uni-rostock.de (A.W.)

**Keywords:** vomeronasal organ, immunohistochemistry, cyclodextrin, allopregnanolone, miglustat, Niemann-Pick disease type C1, mouse model, neurodegeneration, bromodeoxyuridine, olfactory marker protein, cathepsin-D

## Abstract

Introduction: Olfactory impairment is one of the earliest symptoms in neurodegenerative disorders that has also been documented in Niemann-Pick disease type C1 (NPC1). NPC1 is a very rare, neurovisceral lipid storage disorder, characterized by a deficiency of *Npc1* gene function that leads to progressive neurodegeneration. Here, we compared the pathologic effect of defective *Npc1* gene on the vomeronasal neuroepithelium (VNE) with that of the olfactory epithelium (OE) in an NPC1 mouse model. Methods: Proliferation in the VNE and OE was assessed by applying a bromodeoxyuridine (BrdU) protocol. We further compared the immunoreactivities of anti-olfactory marker protein (OMP), and the lysosomal marker cathepsin-D in both epithelia. To investigate if degenerative effects of both olfactory systems can be prevented or reversed, some animals were treated with a combination of miglustat/allopregnanolone/2-hydroxypropyl-cyclodextrin (HPβCD), or a monotherapy with HPβCD alone. Results: Using BrdU to label dividing cells of the VNE, we detected a proliferation increase of 215% ± 12% in *Npc1*−/− mice, and 270% ± 10% in combination- treated *Npc1*−/− animals. The monotherapy with HPβCD led to an increase of 261% ± 10.5% compared to sham-treated *Npc1*−/− mice. Similar to the OE, we assessed the high regenerative potential of vomeronasal progenitor cells. OMP reactivity in the VNE of *Npc1*−/− mice was not affected, in contrast to that observed in the OE. Concomitantly, cathepsin-D reactivity in the VNE was virtually absent. **Conclusion:** Vomeronasal receptor neurons are less susceptible against NPC1 pathology than olfactory receptor neurons. Compared to control mice, however, the VNE of *Npc1*^−/−^ mice displays an increased neuroregenerative potential, indicating compensatory cell renewal.

## 1. Introduction

Niemann-Pick disease type C1 is a rare autosomal-recessive lipid storage disorder with neurodegenerative and neurovisceral phenotypes [1]. The reason is a mutation of the *Npc1* gene on chromosome 18q11 that encodes a transmembranous transport protein with late endolysosomal localization [2,3] responsible for the release of cholesterol from lysosomes [4]. The resulting accumulation of cholesterol, however, is found predominantly in viscera like liver or spleen [5,6,7,8,9], whilst in cells of the nervous system, a different pathologic lipid pattern prevails, consisting of e.g., gangliosides GM2 and GM3 [5,10]. The range of symptoms reaches from hepatosplenomegaly and pulmonary ventilation disorders to neurologic/psychiatric symptoms such as ataxia, kataplexia, seisures, depression, progressive dementia, and retarded language development [11,12,13]. First symptoms may occur as early as 2–3 months after birth, or, in a later form, in later adulthood [14].

We used a mouse model with a BALB/c genetic background that shows similarities in the clinical course of the severe infantile form of the disease. This animal, BALB/cNctr-*Npc1^m^*^1*N*^/J, has a retrotransposon insertion into the N-terminus of the *Npc1* gene, along with a 703-bp deletion, causing premature termination of the coding region that excludes most of the sterol-sensing domain [15]. Whereas motor systems and visceral organs are well studied, there are fewer data available for sensory systems such as vision [16,17], hearing [18,19], or, specifically, chemosensory systems such as olfaction and vomeronasal perception. Within the nervous system, olfactory receptor neurons (ORN) are unique in that they are able to continuously regenerate throughout life [20]. Olfactory neurogenesis is a carefully regulated chain of molecular events that leads to appropriate proliferation and differentiation of basal/precursor cells in the olfactory mucosa [20,21,22]. Since olfactory acuity has been shown to be impaired in several neurodegenerative diseases such as Parkinson’s disease [23,24,25], we previously studied the sense of olfaction in NPC1 and noted severe structural, electrophysiological, and behavioral deficits [26,27]. These alterations included considerable loss of ORN, the presence of autophagosomes, cathepsin-D (CathD)-positive lysosomal inclusions in all mucosal cells, and massive astrogliosis and microgliosis in the olfactory bulb. These findings were accompanied by a 45% increase of BrdU(+) proliferating cells in the olfactory mucosa, compared to wildtype controls [28]. In spite of this considerable attempt of proliferative compensation, the animals performed worse in a buried pellet test than normal controls [27].

Therapeutic options for NPC1 are limited, as there is no causal treatment of the disease. Miglustat, introduced by Lachmann and Platt in 2001 [29] and approved for NPC1 treatment in Europe, Canada, and Japan, is an inhibitor of glucosylceramide transferase [30]. Combined with allopregnanolone, a neuroprotective agent solubilized in 2-hydroxypropyl-β-cyclodextrin (HPβCD) [31], it proved to be effective in animal models and humans [27,28,32,33]. Later, it was discovered that the solvent, HPβCD, was immediately responsible for the cholesterol-reducing effect (reviewed by [34]).

We previously showed that survival of olfactory cells was increased and neurodegeneration prevented upon treatment with HPβCD and/or a substrate reduction therapy with miglustat/HPβCD/allopregnanolone [27,28]. This investigation aims to study if similar alterations also occur in a related chemosensory system, the neuroepithelium of the vomeronasal organ of *Npc1*^−/−^ mice, and how it reacted upon treatment. As the proliferative activity can be regarded as a compensatory reaction of the highly-plastic system to address cell damage, we used a BrdU protocol to determine the proliferative activity of the vomeronasal epithelium. Further, we evaluated the lysosomal activity by immunohistochemical reaction of CathD, and the integrity of vomeronasal receptor neurons with olfactory marker protein (OMP).

## 2. Results

### 2.1. Histology of the Vomeronasal Epithelium (VNE)

**Figure 1:**
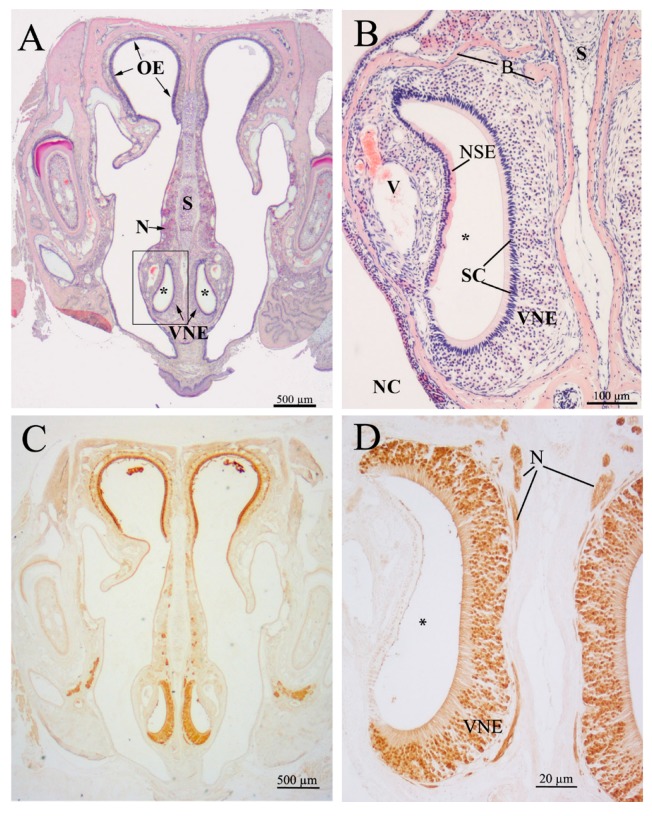
Structural overview of the vomeronasal organ. (**A**) H&E-stained frontal section of the nasal cavity of a *Npc1*^+/+^ mouse, day 57, with the bilateral vomeronasal duct located in the ventral nasal septum. The vomeronasal duct (*) is lined by the receptor-bearing sensory neuroepithelium (VNE). The vomeronasal nerve (N) transfers sensory information along the nasal septum (S) to the accessory olfactory bulb in the telencephalon (not shown). OE, olfactory epithelium; (**B**) minute structure of the vomeronasal duct (*), the VNE, the receptor-free non-sensory epithelium (NSE), and blood vessels (V). S, nasal septum; SC, supporting (sustentacular) cell layer; B, surrounding bone clip; (**C**) immunohistochemical reaction of olfactory marker protein (OMP). OMP identifies olfactory (dorsal) and vomeronasal (ventral) receptor neurons; (**D**) detailed lamination of the pseudostratified VNE. Vomeronasal receptor cells are reactive to OMP; supporting cells (SC) and the opposite, lateral non-sensory epithelial cells are OMP-negative.

**Figure 2:**
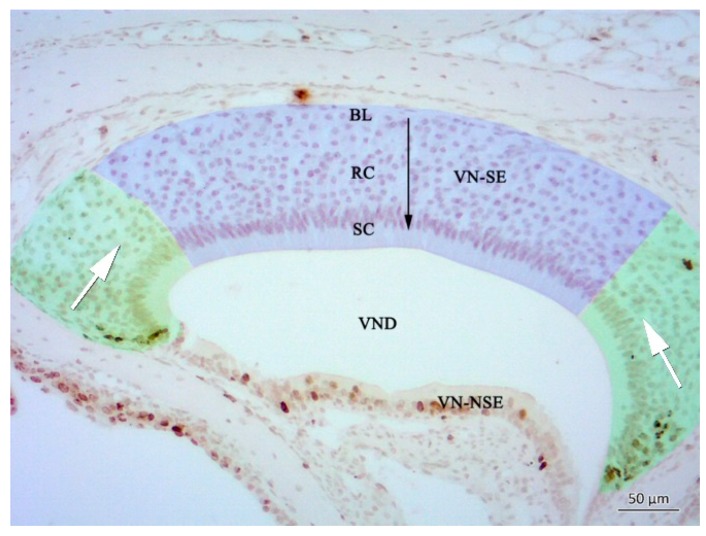
Frontal section of the vomeronasal organ displaying two distinct proliferation zones. Cells of the marginal compartments (green) migrate horizontally (white arrows); cells of the central compartment (blue) migrate vertically (black arrow) thereby following a basal-apical migration route. BL: Basal lamina; RC: nuclei of vomeronasal receptor cells; SC: layer of supporting cells; VN-SE: vomeronasal sensory epithelium, VN-NSE: vomeronasal non-sensory epithelium; VND: vomeronasal duct.

**Figure 3:**
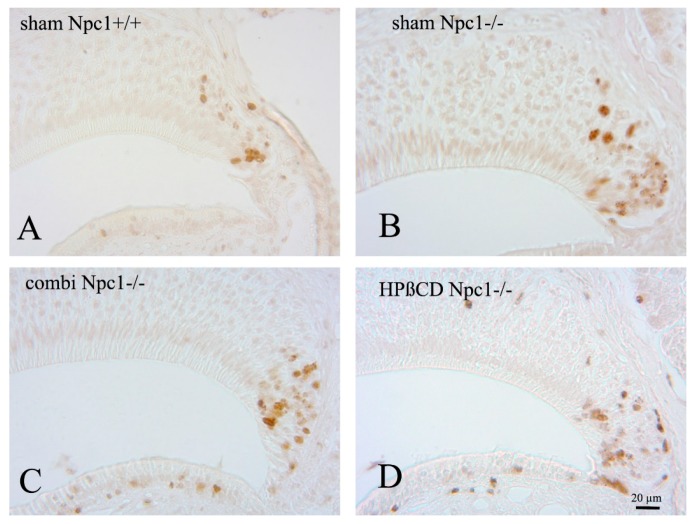
BrdU immunoreactivity of sham-treated *Npc1*^+/+^ (**A**), sham-treated *Npc1*^−/−^ (**B**), combination-treated *Npc1*^−/−^ (**C**) and HPβCD-treated *Npc1*^−/−^ (**D**) animals. Whereas the structural integrity is maintained in all groups, the number of BrdU(+) cells is clearly increased in all mutant animal groups. (**A**) Most labeled nuclei belong to cells located in the boundary region between neuroepithelium and supporting cell layer; (**B**–**D**) mutant animals house BrdU(+) equally in all three layers (basal, intermediate and apical). Treated mutant mice also have increased cell numbers in the non-sensory epithelium.

**Figure 4:**
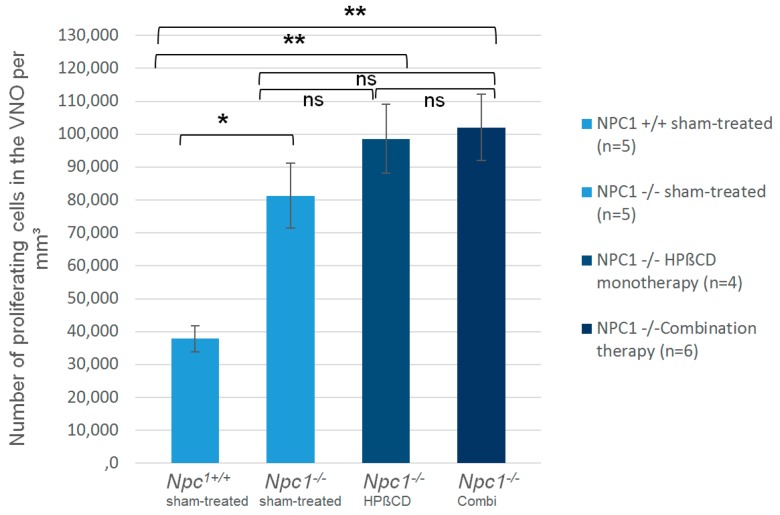
BrdU(+) cells within the vomeronasal epithelium of mutant and treated mice show a significant increase of proliferating cells in sham-treated *Npc1*^−/−^ mice and both, HPβCD-treated and combination treated animals in comparison to sham-treated *Npc1*^+/+^ animals. * *p* < 0.05; ** *p* < 0.01; for *p* values see text.

**Figure 5:**
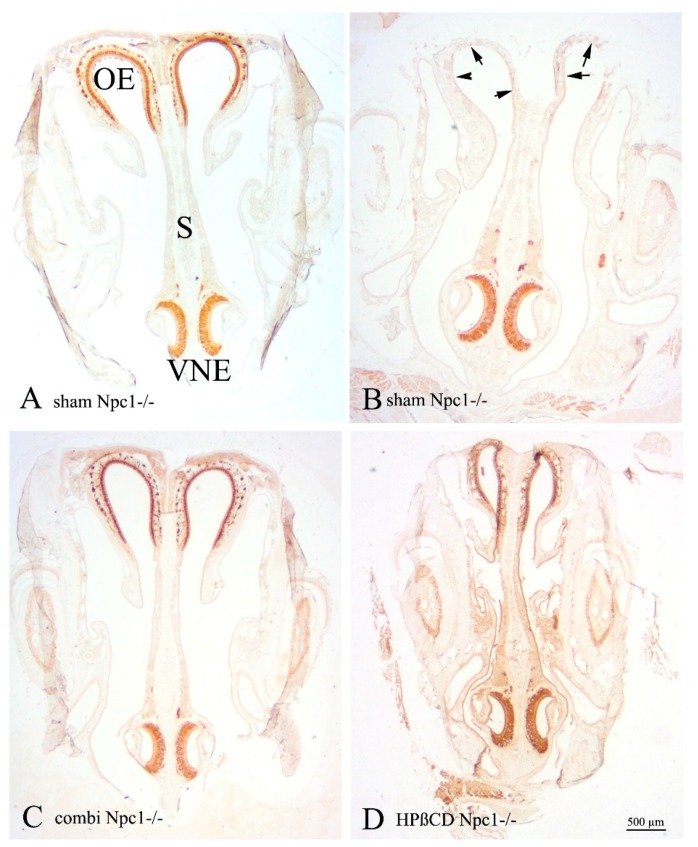
Comparison of OMP immunoreactivity (IR) in both nasal chemosensory organs. (**A**) OMP-IR in sham-treated *Npc1*^+/+^ mice is regularly distributed in olfactory receptor neurons (OE) and in vomeronasal receptor neurons (VNE); (**B**) sham-treated *Npc1*^−/−^ mice present highly damaged and almost OMP-IR absent neurons within the OE (arrows), whereas the VNE appears normal; (**C**,**D**) both treated animal groups, however, show almost normal OMP-IR indicating a protective effect of both treatment strategies. The VNE does not show changes of OMP-IR in neither condition.

**Figure 6:**
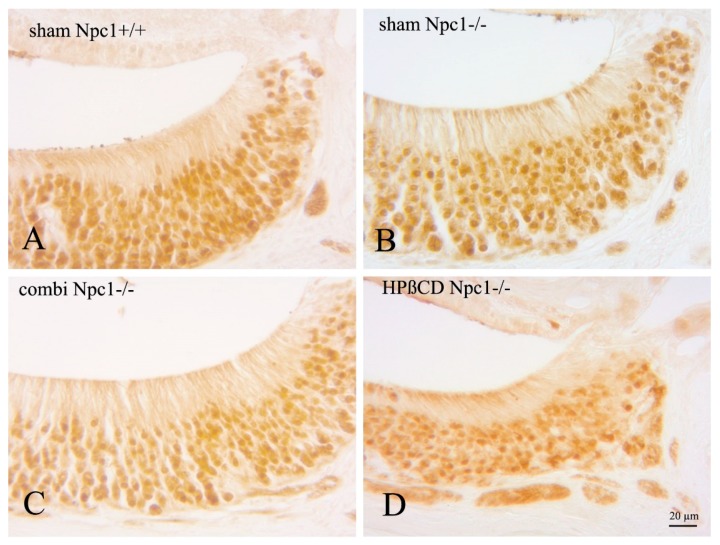
Detailed images of OMP immunoreactivity (IR) of vomeronasal epithelial cells in *Npc1*^+/+^ (**A**) and *Npc1*^−/−^ mice (**B**–**D**). Intensities and distribution of OMP-IR do not apparently differ within the groups.

The VNE lines a blind-ending bilateral semilunar duct situated in the ventral nasal septum (Figure 1). The duct consists of a lateral, non-sensory and a medial, sensory epithelium. In the latter, basal precursor cells are not arranged in a distinct cell layer, like in the OE, but occur basally, extending from the anterior and posterior margins to the central area over the entire epithelium. Differentiating sensory cells occupy the largest volume within the epithelium. The apical surface of the vomeronasal duct is lined by supporting (sustentacular) cells, and intercalated dendrites of vomeronasal receptor cells, which are situated in the middle and basal third of the epithelium, similar to the OE (Figure 1 and Figure 2). We did not find major differences in the overall histological structure of the VNE between groups (Figure 3, Figure 4, Figure 5 and Figure 6).

### 2.2. Quantification of BrdU(+) Proliferating Cells

Most BrdU(+) proliferating cells were located in the anterior and posterior edges of the semilunar-shaped VNE (Figure 2 and Figure 3), giving rise to a horizontally-oriented way of cell migration (green area in Figure 2; see also [35,36]). A minor proliferative compartment exists in the basal layer of neuroepithelial precursors that migrate in a more vertical pathway (blue area in Figure 2) [35,36]. The former can be considered as a “pool for replacement”, and the latter as a “pool for growth” (Figure 2) [35].

In order to evaluate the proliferation of vomeronasal epithelial cells, we quantified BrdU-labelled cells within the VNE of the left side, since previous investigations had not shown any side differences. Morphometric studies in rats did not observe any sex-related differences [35]. We defined the number of BrdU(+) cells per mm^3^ of sham-treated Npc1^+/+^ 100% with 37,827 (±3907) cells/mm^3^, and compared the cell densities. The proliferation in all other groups was significantly higher than in the control group (Figure 4; Table 1). The cell number in sham-treated *Npc1*^−/−^ animals was significantly elevated by 215% (81,287 ± 9900; *p* < 0.014). Both treatments led to a significant increase of cell densities compared to *Npc1*^+/+^ control animals, but not to sham-treated *Npc1*^−/−^ mice. The number of BrdU(+) cells in combination-treated *Npc1*^−/−^ animals significantly exceeded the level of Npc1^+/+^ mice by 270% (102,080 ± 10.097, *p* < 0.006). Monotherapy with HPβCD led to a significant increase of cell density compared to Npc1^+/+^ mice (98,626 ± 10,388/ mm^3^, *p* < 0.014), but this was not significant compared to sham-treated *Npc1*^−/−^ mice (*p* > 0.221). Quantification of OE was published previously [28] (Table 1, right column). In the OE the density of BrdU(+) cells was lower than in the VNE, but treatments led to a significantly higher proliferation in the OE.

### 2.3. Caspase-3(+) Immunohistochemistry

Cleaved caspase-3 (cas3) antibodies were used to estimate the extent of apoptosis in the VNE [37]. Most apoptotic cells were found in the middle compartment (Supplementary Figure S1), which is in accordance with observations of De la Rosa-Prieto and co-workers [37]. Differences between the groups were not observed. For quantification, the number of cells was too small (0–1) per section to achieve informative values.

### 2.4. OMP(+) Mature ORNs and Vomeronasal Receptor Neurons

Olfactory marker protein (OMP) detects mature olfactory receptor neurons (ORNs), and is involved in the olfactory signal transduction pathway [38,39,40]. It has also been described as a marker for vomeronasal receptor cells in various animals [41,42,43]. OMP bound to cytoplasmic epitopes of dendrites, and axons of the vomeronasal nerve, as well as to nuclear binding sites. OMP(+) cells were regularly distributed in the VNE of sham-treated *Npc1*^+/+^ control animals, occupying all layers of the sensory epithelium. Although not quantified, there was no apparent qualitative change of distribution and density of OMP(+) cells, compared to all mutant mice investigated in this study (Figure 5). However, OMP(+) nuclei of *Npc1*^−/−^ mice, both with and without treatment, showed a flattened morphology (Figure 6). The most remarkable intraindividual difference between both chemosensory epithelia was the severe morphological alteration of the OE in sham-treated *Npc1*^−/−^ mice compared to that of the VNE.

### 2.5. Cathepsin D Immunohistochemistry

**Figure 7:**
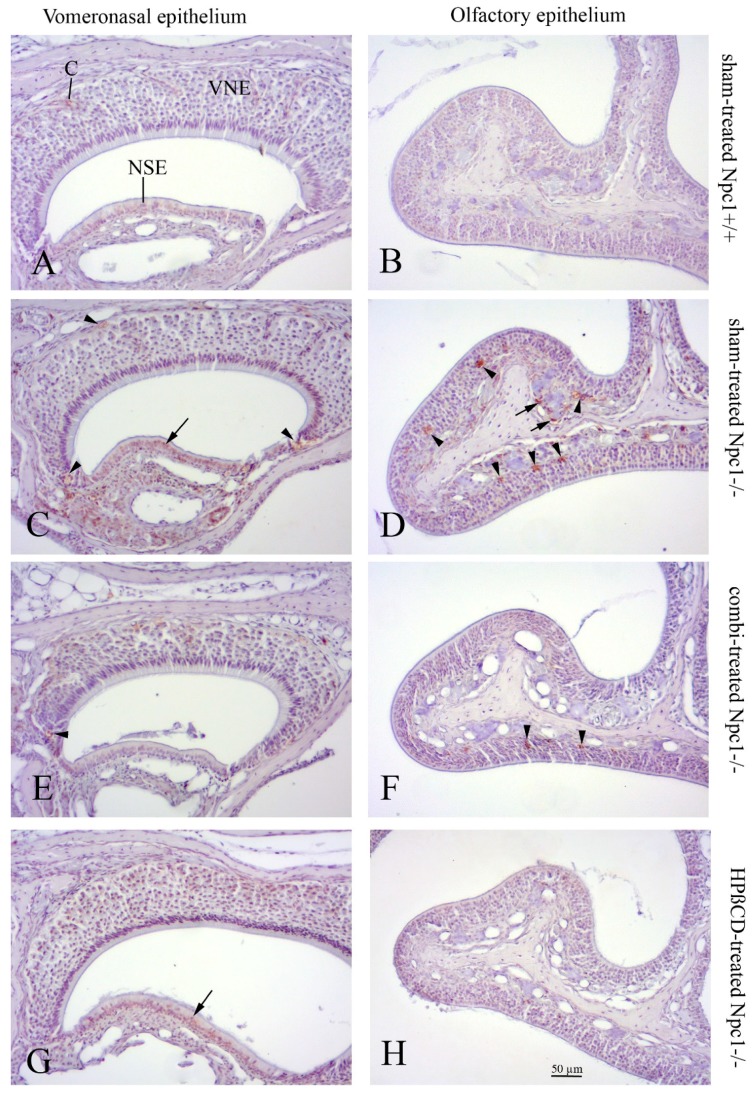
Cathepsin D (Cath D)-immunohistochemical comparison of lysosomal activity between vomeronasal (left column) and olfactory mucosa (second endoturbinate; right column) of the correspondent animals. (**A**) Apart from a slight positive reaction of non-sensory epithelium (NSE) and intraepithelial capillaries (c), sham-treated *Npc1*^+/+^ mice show almost no Cath D(+) cells; (**B**) almost no reaction in the OE of sham-treated *Npc1*^−/−^ animals; (**C**) sham-treated *Npc1*^−/−^: Cath D(+) reaction in proliferative marginal zones (arrowheads), NSE (arrow) and underlying connective tissue, but barely within the VNE. (**D**) In contrast, the same animal presents numerous Cath D(+) lysosomes in the OE, probably of macrophages (arrowheads); also within olfactory ensheathing cells around olfactory nerve fiber bundles in the lamina propria (arrows). Combination-treated *Npc1*^−/−^ mice (**E**,**F**) and HPβCD-treated *Npc1*^−/−^ (**G**,**H**) animals presented a somewhat changed nuclear morphology (flattened nuclei), but markedly reduced Cath D-reactive deposits (arrow in **G**; arrowheads in **F**) indicating that treatment did not completely prevent pathologic alterations.

Cathepsin D (CathD) was used to detect lysosomes that usually react strongly positively in ORN and macrophage-like cells within the OE of sham-treated *Npc1*^−/−^ animals [28]. In contrast, CathD immunoreactivity was almost absent in sham-treated *Npc1*^+/+^ animals. Some faint, if any, CathD reactivity occurred in cells located in the VNE edges. A difference between the CathD immunoreactivities was not apparent in the treatment groups (Figure 7).

## 3. Discussion

In this report, we compared the proliferative behavior of vomeronasal epithelial cells with cells of the main OE in NPC1. A main finding of this study was enhanced proliferation of VNE cells in all *Npc1*^−/−^ groups, however, to a much lesser degree than in cells of the OE. Accordingly, the OMP reactivity was maintained in the VNE, whereas it appeared to be severely disrupted in the OE (detailed data of the OE were previously reported [28]).

### 3.1. Vomeronasal vs. Main Olfactory Epithelium

Chemosensation in rodents relies on a subset of diverse structures, namely the classical olfactory mucosa, the vomeronasal organ (VNO), the septal organ of Masera [44,45], and the Grüneberg ganglion [46,47]. The VNO (organ of Jacobson) is an accessory olfactory organ that receives chemical stimuli in order to elicit neuroendocrine, behavioral or reproductive responses among individuals of the same species [41,48,49,50,51]. In many species, it constitutes a complex system including the VNE, the vomeronasal nerve, and the first CNS relay, the accessory olfactory bulb. Canonical neuroanatomical pathways are well described for main olfactory and vomeronasal perception. Structural similarities are OMP(+) neuroepithelial cells and overall principles of the G-protein-coupled signaling cascade. Vomeronasal receptors, however, are unrelated to olfactory receptors and integrated in membranes of microvillar processes belonging to apical (V1R) or basal (V2R) vomeronasal receptor cells [52]. Furthermore, they project to different glomeruli of the accessory OB.

### 3.2. VNE Proliferation is Enhanced in Npc1^−/−^ Mice, But Without Signs of Degeneration

The proliferation of OE cells in sham-treated *Npc1*^−/−^ mice may be understood as a compensation mechanism for the dramatic loss (approx. 40%) of olfactory neurons [28,53]. Although not fully balanced by respective numbers of apoptotic cas-3(+) cells, the reduced number of OMP(+) cells can be explained by the failure of the olfactory progenitor/basal cells to compensate for the high number of dying ORNs [28,53]. Under normal conditions, the fate of newly-born cells in the VNE is either maturation into OMP(+) cells or apoptosis, the extent of which is dependent on the location within the epithelium. In the marginal compartments, the maturation prevails, whereas in the central compartment neurogenesis and apoptosis, it is balanced [37]. However, it must be considered that neurogenesis declines steeply with age [35], and relevant cell renewal in the central VNE may not be seen after P56, when our data were collected.

Hence, the increased proliferation in the VNE is neither accompanied by enhanced apoptosis in sham-treated *Npc1*^−/−^ mice, nor by reduction in OMP reactivity. This indicates (1) a proliferative force being independent of degeneration phenomena as hypothesized for the OE, and (2) a less sensitive nature of mature vomeronasal epithelial cells towards the pathologic effects of *Npc1* mutation. The latter is supported by the virtual absence of Cath D inclusions in VNE cells, whereas OE cells contained numerous Cath D(+) plaques, a sign of enhanced lysosomal activity typically seen in NPC1 [33,54,55] and other neurodegenerative disorders [56,57].

### 3.3. Distinct Differences of Regeneration Mechanisms in OE and VNE

What is more, both epithelia are different in terms of cell replacement mechanisms, as the OE reconstitutes from vertical maturation and differentiation of basal cells, whereas regeneration of the VNE is initiated in the margins of the epithelium, leaving the horizontal areas largely empty of newborn neurons [35,36]. Weiler et al. [35] argued that after termination of growth in adults, newly-generated cells were quickly eliminated, keeping the effective rate of neuronal turnover low and not replacing existing neurons.

Therefore, it can be assumed that cells in the central, almost BrdU-negative compartment (“pool for growth”, [35]) have a longer life span than cells of the “pool of replacement” in the margins of the VNE (see Figure 2). Also, traces of lysosomal Cath D activity, if visible at all, occur in these compartments of regeneration (arrowheads in Figure 7E). It can be speculated that, at least in the VNE, proliferative tissues are more responsive to NPC1-specific cell pathology than non-proliferative ones. Our own observations in visceral organs in this *Npc1* mouse model indicate that tissues are differently vulnerable; e.g., cardiac muscle, apart from endothelium, shows almost no inclusions of lipid substances in autophagosomes, whereas lung, liver, and the CNS are much more susceptible. In the olfactory bulb, astroglia and oligodendroglia tend to accumulate earlier and more myelin-like deposits than neurons [33]. However, the minute nature of reasons leading to different tissue pathophenotypes in NPC1 remains unclear. Destructive tissue integration at the levels of OE and olfactory bulb also led to reduced olfactory performance in a simple olfactory screening test [27], but the more complex, pheromone-induced behavioral consequences in *Npc1* animals were not yet tested.

### 3.4. Treatment: Both Strategies Do Increase Proliferation, But Do Not Change the VNE Phenotype

NPC1 remains a fatal disease, as there exists no causal therapy option. However, plenty of data illustrate the beneficial effects of current symptomatic treatments. The combination of miglustat, HPβCD and allopregnanolone [27,28,32,33,58] leads to effective prevention of neurologic symptoms, and also reduced hepatopathology [6,59]. HPβCD seems to be the effective agent [9,34] by directly replacing the function of NPC proteins within endosomal and lysosomal compartments [60,61]. The toxic potential of this drug is apparently low, as revealed in a range of concentrations comparable to those applied in this study [9,62]. The only major adverse effect in human patients consists of hearing loss, apparently caused by toxic interaction of HPβCD with outer hair cells [63,64,65]. The main olfactory system, both central and peripheral portions, respond well to therapeutic intervention, i.e., the OE barely showed pathologic cell inclusions or ORN loss [28], as well as reduced CNS inflammatory events, normalized olfactory gene expression patterns and behavior tests [27,53]. These significant differences were, of course, not documented in the VNE, for the simple reason that dramatic pathologic changes had not occurred. The only hint of a pharmacologically-induced alteration is the increased proliferation rate, but, being lower than that of the OE, even this did not reach significant values compared to sham-treated *Npc1*^−/−^ mice.

## 4. Methods

### 4.1. Animals

Heterozygous breeding pairs of *Npc1* mice (BALB/cNctr-*Npc1*m1N/-J) were obtained from Jackson Laboratories (Bar Harbor, ME, USA) for generating homozygous *Npc1*^−/−^ mutants and *Npc1*^+/+^ control wild type mice. Mice were maintained under standard conditions with free access to food and water with a 12 h day/night cycle, a temperature of 22 °C and a relative humidity of 60%. Genotypes were determined until postnatal day P7 by PCR analysis. 15 *Npc1*^−/−^ mutants and 5 wild type controls of both sexes, aged to 8 weeks, were used for different therapeutic treatment schedules.

This study was carried out in accordance with the recommendations of the German legislation on protection of animals and the Committee on the Ethics of Animal Experiments at the University of Rostock. The protocol was approved by the Landesamt für Landwirtschaft, Lebensmittelsicherheit und Fischerei Mecklenburg-Vorpommern (approval ID: 7221.3-1.1-030/12, 14 June 2012).

### 4.2. Genotyping

For genotyping by PCR analysis, 1–2 mm of the tails were clipped at postnatal day P6 and homogenized in DirectPCR-Tail and 1% proteinase K (Peqlab, Erlangen, Germany) at 55 °C with 750 rpm for 16 h overnight on a Thermo Mixer (Eppendorf, Hamburg, Germany). Extracts were centrifuged for 30 s with 6000 rpm, and PCR analysis was performed twice with 2 µL of the lysate and two different primer pairs under equal cycling conditions. For detecting the mutant allele (obtained fragment size 475 bp) primers 5′-ggtgctggacagccaagta-3′ and 5′-tgagcccaagcataactt-3′ and for the wild type allele (obtained fragment size 173 bp) 5′-tctcacagccacaagcttcc-3′ and 5′-ctgtagctcatctgccatcg-3′ were used.

### 4.3. Pharmacologic Treatment

The following 4 groups were evaluated: (1) Sham-treated *Npc1*^+/+^ (wild type) mice; (2) sham-treated *Npc1*^−/−^ mutant mice; (3) *Npc1*^−/−^ mutant mice, which received a combination therapy; (4) *Npc1*^−/−^ mutant mice, which received a HPßCD monotherapy.

**Figure 8:**
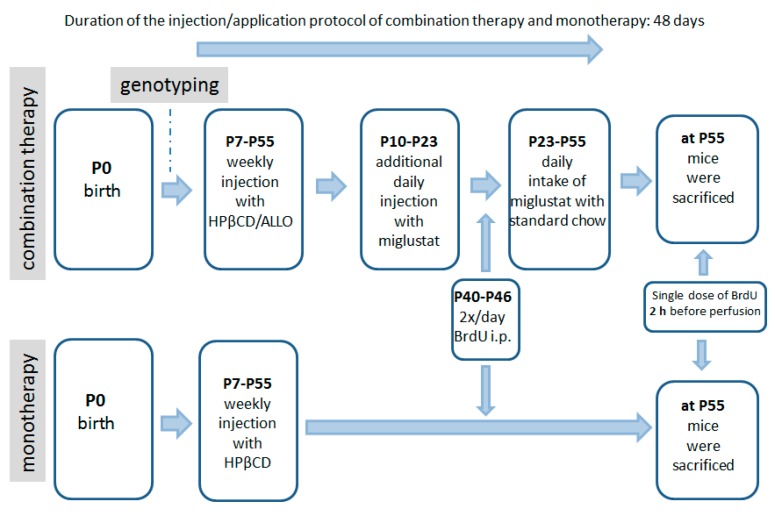
Scheme of the drug application for combination treatment (allopregnanolone, miglustat, HPβCD), the monotherapy with HPβCD and BrdU.

Two different therapeutic schedules for the *Npc1*^−/−^ mutants and their *Npc1*^+/+^ controls were applied: (1) a combination treatment (combi) of synergistically working drugs utilizing HPβCD, allopregnanolone and miglustat, starting at postnatal day P7 with an injection of allopregnanolone (Pregnan-3alpha-ol-20-one; 25 mg/kg; Sigma Aldrich, St. Louis, MO, USA) dissolved in 2-hydroxypropyl-*β*-cyclodextrin (HPβCD); 4000 mg/kg, i.p.; Sigma Aldrich, in Ringer’s solution, once a week, as described earlier [27,28,32]). Additionally, 300 mg/kg miglustat (*N*-butyl-deoxynojirimycin; generous gift of Actelion Pharmaceuticals, Allschwil, Schwitzerland) dissolved in normal saline solution was intraperitoneally injected daily from P10 to P22. Then, miglustat powder was administered mixed with food (summarized in Figure 8). And (2) allopregnanolone and miglustat were omitted and only HPβCD was injected weekly. Controls included treated and sham-treated *Npc1*^+/+^ animals as well as sham-treated *Npc1*^−/−^ mutants. Sham-treated mice received Ringer solution instead of pharmacologic treatments, but were physically handled in the same way like treated animals.

### 4.4. BrdU Injections

BrdU (5-bromo-2′-deoxyuridine) is a thymidine analogue, which is incorporated in DNA during the S-phase of DNA synthesis. Consequently, it is a reliable marker for the quantification of the proliferative potential of tissues [66,67]. All mice were i.p. injected with BrdU (solubilized in normal saline, 50 mg/kg, Sigma, St. Louis, MO, USA) twice a day from P40 to P46. Additionally, a final single dose was given 1 h before perfusion at P55–56 for labeling the dividing cells of the sensory epithelium.

### 4.5. Sample Preparation

Mice were deeply anesthetized with a mixture of 50 mg/kg ketamine hydrochloride (Bela-Pharm GmbH & Co KG, Vechta, Germany) and 2 mg/kg body weight of xylazine hydrochloride (Rompun; Bayer HealthCare, Leverkusen, Germany), and then intracardially perfused with normal saline solution, followed by 4% paraformaldehyde (PFA) in 0.1 M PBS. Mice were then decapitated, skinned, spare tissue was removed and the remaining skull, including the anterior nasal septum with the VNO, and nasal turbinates were post-fixed in 4% PFA for 24 h at 4 °C. Subsequently, heads were decalcified in 10% EDTA for 5–6 days at 37 °C, dehydrated and embedded in paraffin. The heads were serially cut in 10 µm in frontal direction from the tip of the nose to the anterior olfactory bulb and collected.

### 4.6. Histology and Immunohistochemistry

For routine inspection, sections of the nasal septum were stained with hematoxylin & eosin (H&E) with an interval of 500 µm. For the quantification of proliferating cells every 10th section (spaced 100 µm apart) was subjected to anti-BrdU immunohistochemistry (Abd Serotec, Puchheim, Germany). Sections were deparaffinized, rehydrated, and pretreated with microwaves in 0.1 M citrate buffer (5 min, 680 W), followed by incubation with 3% H_2_O_2_ in PBS to block endogenous peroxidases for 30 min, and 5% normal goat serum (NGS) in PBS for 45 min to block nonspecific binding sites. Subsequently, sections were exposed to the primary antibody against BrdU (1:2000) in 3% NGS/PBS overnight at 4 °C. One section of each slide was used for negative control. After washing in PBS, the sections were sequentially incubated for 1 h with the secondary anti-rat IgG (1:200; Vector, Burlingame, CA, USA), the avidin-biotin-complex (ABC) reagent for 1 h (Vectastain-Elite; Vector), and finally visualized with H_2_O_2_-activated 3,-3,-diaminobenzidine (DAB, Sigma, Munich, Germany). Sections were dehydrated, mounted with DePeX and coverslipped.

For the qualitative evaluation of the VNE and its receptor cells adjacent sections were pretreated with microwaves in 0.1 M citrate buffer (5 min 850 W and 5 min 340 W). After blocking procedures, sections were exposed either to rabbit anti-OMP (1:6000, Cat No. O7889, Sigma, Saint Louis, MO, USA), cas-3 (1:500, clone Asp175, Cat No. 9661, Cell Signaling Technology, Danvers, MA, USA), or anti-cathepsin D (1:6000, Cat No. PU205-UP, BioGenex Laboratories, San Ramon, CA, USA) overnight at 4 °C and subsequently developed as described above. For controls, primary antisera were omitted. In negative controls, no reactivity was observed.

### 4.7. Stereology and Statistic Evaluation

For quantification, BrdU-positive cells of the VNE in 4–5 sections of each mouse were counted, using an unbiased stereological method: the optical fractionator [68]. The vomeronasal duct and the lateral non-sensory epithelium were excluded. For each group and each genotype, 4–6 animals were counted using a computer-aided microscope (Olympus BX-51, Hamburg, Germany) and stereology software (Stereo Investigator v7.5, MBF Bioscience, Williston, ND, USA). The whole VNE was first outlined using a 2× or 4× objective lens. Counting was realized at 40× magnification. The cell density of proliferating cells per mm^3^ of VNE was averaged, and the four different groups (summarized in Table 1) were compared. Therefore, the sham-treated mutants served as a reference for both combination-treated and HPβCD-treated mice.

Results are expressed as mean values ±SEM. Statistical evaluation was done with a Mann-Whitney U-test by SPSS (v.15.0.1, Chicago, IL, USA) using genotype and treatment group as independent variables. *p* < 0.05 was considered significant.

## 5. Conclusions and Hypotheses

The vomeronasal neuroepithelium in mice underlies the mechanisms of replacement/growth which differ from those of the olfactory mucosa. Here, the VNE in *Npc1*^−/−^ mice seems to be less susceptible to pathologic effects of *Npc1* mutation. A possible speculation could be that the VNO constitutes not only an accessory chemosensory organ with a distinct set of receptors and neuroanatomical pathways, but may also rely on main-olfactory-independent alternative security systems that protect in case of any endotoxic (genetic) or exotoxic environmental stress. However, to validate this assumption, a complete set of cell biological, genetic, and behavioral investigations is necessary.

## Figures and Tables

**Table 1:** Summary of the mean values ± SEM in cells/mm^3^ of the cell densities of proliferating BrdU(+) cells in the vomeronasal epithelium.

Animal Group	VNE Cells/mm³ ± SEM		OE * Cells/mm³ ± SEM	
sham-treated *Npc1*^+/+^	37,827 ± 3,907		17,417 ± 1,317	
sham-treated *Npc1*^−/−^	81,287 ± 9,900	+215%	25,180 ± 1,605	+144%
HPβCD-treated *Npc1*^−/−^	98,626 ± 10,388	+260%	44,693 ± 4,191	+257%
Combination-treated *Npc1*^−/−^	102,080 ± 10.097	+269%	70,558 ± 8159	+405%

The percentages indicate the increase relative to the number of sham-treated Npc1^+/+^ mice. * Data of the OE were assessed previously in the same animals [28].

## Supplementary Materials

Supplementary materials can be found at http://www.mdpi.com/1422-0067/19/11/3563/s1.

## Abbreviations

The following abbreviations are used in this manuscript:

BrdU	5-bromo-2′-deoxyuridine
cas-3	cleaved caspase-3
Cath D	cathepsin D
HPβCD	2-hydroxypropyl-β-cyclodextrin
*Npc1*	*NPC1 gene*
NPC1	NPC protein 1
OE	olfactory epithelium
OMP	olfactory marker protein
ORN	olfactory receptor neuron
VNE	vomeronasal epithelium
VNO	vomeronasal organ
