# Supplementary figures and images for: Main Olfactory and Vomeronasal Epithelium Are Differently Affected in Niemann-Pick Disease Type C1
Source: Int J Mol Sci. 2018 Nov 12;19(11):3563. doi: 10.3390/ijms19113563 (PMC6274921; doi:10.3390/ijms19113563)

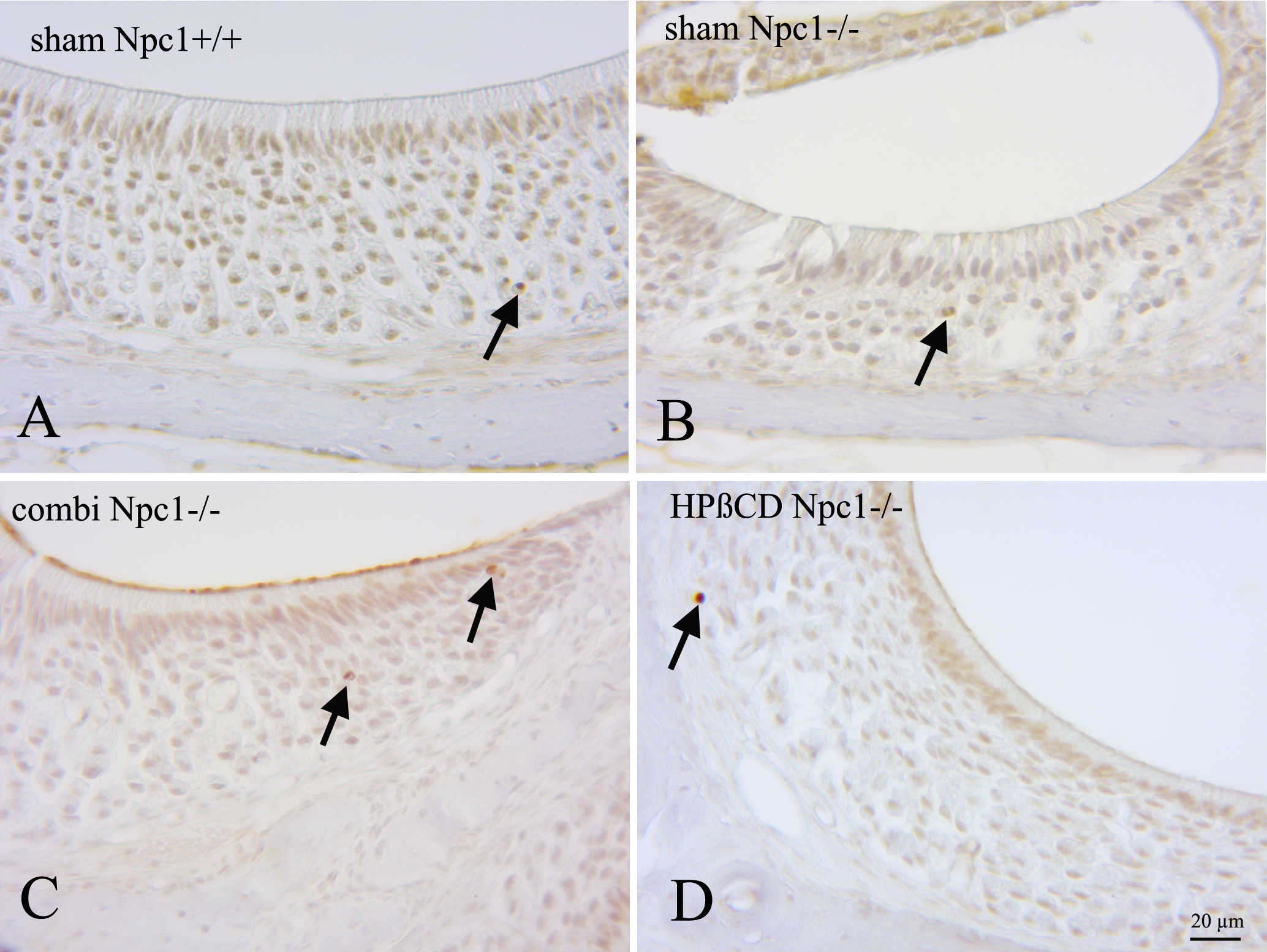

Supplement: Supplementary file 1 [file ijms-19-03563-s001.zip › ijms-373836-supplementary.tif]
